# Chronic Alcohol Consumption Reprograms Osteoclast Lineage Communications to Promote Osteoclastogenesis

**DOI:** 10.3390/biology15070527

**Published:** 2026-03-26

**Authors:** Hami Hemati, Brianna M. Doratt, Ilhem Messaoudi

**Affiliations:** Microbiology, Immunology and Molecular Genetics, College of Medicine, University of Kentucky, Lexington, KY 40536, USA; brianna.doratt@uky.edu

**Keywords:** chronic alcohol consumption, non-human primates, osteoclastogenesis, osteoclast precursors, osteoclast, CellChat

## Abstract

Chronic alcohol consumption increases the risk of osteoporosis and fracture by disrupting bone remodeling, in part by enhancing osteoclastogenesis. However, the cellular mechanisms underlying this process remain incompletely defined. We analyzed scRNA-seq data from osteoclasts differentiated in vitro from bone marrow mononuclear cells obtained from macaques following 12 months of chronic ethanol or isocaloric control solution consumption. Module scoring, trajectory inference with generalized additive modeling (tradeSeq), and CellChat-based analyses of intercellular communication were applied to uncover ethanol-induced changes in metabolic reprogramming, lineage progression, and signaling network dynamics. Module scoring indicated metabolic reprogramming toward oxidative phosphorylation, with reduced glycolytic, migratory, and phagocytic activities. Pseudotime analysis revealed accelerated osteoclast lineage commitment, broader intermediate differentiation states, and stabilization of mature osteoclasts. CellChat analysis showed globally amplified intercellular signaling, with mature osteoclasts functioning as dominant communication hubs sustained by autocrine feedback. Together, chronic alcohol consumption rewired osteoclastogenesis through early fate priming, metabolic adaptation, and hierarchical remodeling of intercellular communication, promoting enhanced osteoclastogenesis. These findings provide mechanistic insight into alcohol-induced bone pathology and highlight potential targets for therapeutic intervention.

## Simple Summary

Chronic alcohol consumption is a major risk factor for bone loss, yet the cellular mechanisms driving alcohol-induced osteoporosis remain poorly understood. Bone remodeling relies on a delicate balance between osteoblast and osteoclast activities that regulate bone formation and resorption, respectively. Osteoclasts originate from macrophage-lineage precursors in the bone marrow. Using single-cell RNA sequencing (scRNA-seq), we investigated how alcohol alters osteoclast differentiation and communication among bone marrow-derived cells. We found that alcohol reprogrammed cellular metabolism, favoring oxidative phosphorylation and reducing phagocytic activity during osteoclast maturation. We further found that alcohol accelerated early osteoclast lineage commitment through amplified signaling networks. Mature osteoclasts acted as dominant signaling hubs maintained primarily through enhanced developmental, adhesion, and survival signaling. Together, these findings indicate that chronic alcohol misuse enhances osteoclastogenesis through early fate priming, metabolic adaptation, and altered intercellular communication of the osteoclast lineage. These mechanistic insights identify potential therapeutic targets for preventing alcohol-induced bone loss.

## 1. Introduction

In the United States, among over 134.3 million people ages 12 and older who reported drinking in the past month, 14.5 million reported heavy alcohol use in 2024 [1]. Heavy chronic alcohol consumption is strongly associated with decreased bone mineral density and an increased risk of fractures [2,3,4]. Consumption of 2 or more drinks per day increases the risk of osteoporosis [3], and 3 or more drinks per day elevates the risk of hip fractures [4]. Such skeletal complications arise from the systemic effects of alcohol across multiple organs, which directly and indirectly disrupt bone homeostasis. In particular, alcohol directly affects cell populations involved in bone development, including osteoclasts and osteoblasts, as well as hematopoietic stem and progenitor cells (HSPCs) [5] and mesenchymal stem cells [6,7].

In postnatal life, osteoclasts are derived from the monocyte/macrophage compartment of HSPCs [8,9]. Differentiation of monocyte/macrophage precursors into osteoclasts involves membrane raft assembly, proliferation, RNA metabolism, and energy metabolism. This process is initiated primarily through RANK/RANKL and M-CSF/CSF1R signaling, followed by nuclear translocation of transcription factors that drive expression of essential osteoclast genes such as *DC-STAMP*, *MMP9*, *CTSK*, and *ACP5*, culminating in osteoclast fusion and functional maturation [8,9,10,11,12]. However, the magnitude of differentiation, as well as the number and activity of mature osteoclasts, are regulated by a complex network of additional signaling pathways, including both intracellular and intercellular mediators.

HSPCs are highly sensitive to alterations within the bone marrow microenvironment. Indeed, we have previously demonstrated that chronic alcohol consumption in a non-human primate model skews HSPCs differentiation toward granulocyte–monocyte progenitors [5,13] while concurrently enhancing their osteoclastogenic capacity [5]. Previous studies using this model reported skeletal alterations, including suppression of intracortical bone remodeling and reduced cancellous bone formation [14,15,16,17,18]. Although we and others have shown that alcohol dysregulates osteoclastogenesis, the underlying mechanisms remain incompletely understood. In particular, the contribution of interlineage regulators remains undefined.

Therefore, to gain a deeper understanding of the alcohol-mediated mechanisms regulating osteoclastogenesis, we leveraged access to a single-cell transcriptomic dataset generated from in vitro differentiation of bone marrow cells from non-human primates that had been chronically consuming alcohol and from controls into osteoclasts [5]. Our analysis revealed chronic alcohol consumption rewires osteoclastogenesis through early fate priming, metabolic adaptation, and remodeling of intercellular communication. These mechanistic insights identify potential therapeutic targets for preventing alcohol-induced bone loss.

## 2. Materials and Methods

### 2.1. Study Design

This study used a scRNA-seq dataset from SRA# PRJNA1223457 [5]. Briefly, this dataset was generated from bone marrow mononuclear cells obtained from eight control rhesus macaques (4 females, 4 males) and nine macaques (5 females, 4 males) that engaged in very heavy voluntary alcohol consumption throughout a 12-month period [19,20,21]. The cells were differentiated into osteoclasts in the presence of macrophage colony-stimulating factor (M-CSF) (PeproTech, Rocky Hill, NJ, USA) and receptor activator of nuclear factor κB ligand (RANKL) (PeproTech). Following differentiation, cells were harvested, labeled with hashtag oligonucleotides (BioLegend, San Diego, CA, USA), pooled, and loaded onto a Chromium Controller (10x Genomics, Pleasanton, CA, USA). Single-cell libraries were prepared using the Chromium Single Cell 3′ v3.1 Kit (10x Genomics) and sequenced at Novogene (Sacramento, CA, USA) on a NovaSeq X Plus platform (Illumina, San Diego, CA, USA).

Sequencing reads were aligned to the rhesus macaque reference genome (Mmul_10) using Cell Ranger (version 7.2), and downstream analyses were performed in R (version 4.1.1) using Seurat (version 5.1). Cells with low transcript detection (<400 genes) or high mitochondrial gene expression (>5%) were excluded from the analysis. The remaining 15,445 cells from all samples were integrated using Harmony (version 1.2), followed by normalization with NormalizeData. Cell clustering was performed using the first 10 principal components by applying the FindNeighbors and FindClusters functions in Seurat, with a resolution of 0.3 [5].

### 2.2. Trajectory Inference and Pseudotime Analysis

Trajectory inference was performed using Slingshot (version 2.7) [22] with the condiments package (version 1.5) [23] to reconstruct developmental lineages and compute pseudotime values for cells along differentiation trajectories. The Seurat object was converted to a SingleCellExperiment object, and trajectory inference was conducted on the Harmony dimensionality reduction with “Differentiating Macrophages” specified as the starting cluster. Slingshot was run with 150 approximation points to construct smooth principal curves representing differentiation paths. Cell imbalance scores across conditions were calculated using the imbalance_score function from bioc2020trajectories with k = 20 neighbors and a smoothing parameter of 40. Trajectory curves were refined using the getCurves function with shrink = TRUE, extend = “n” to exclude outliers, and smoother = “smooth.spline” with default parameters.

### 2.3. Differential Gene Expression Along Trajectories

Differential expression analysis along pseudotime trajectories was performed using tradeSeq (version 1.8) [24]. The top 2000 highly variable genes identified by Seurat were used as input features. Generalized additive models (GAMs) were fitted using the fitGAM function to model gene expression as a smooth function of pseudotime for each lineage. For condition-based comparisons between control and alcohol groups, the conditionTest function was applied with a log2 fold-change threshold of 2 (l2fc = log2(2)) to identify genes with significantly different expression patterns between conditions along each trajectory. Multiple testing correction was performed using the Benjamini–Hochberg false discovery rate (FDR) method, and genes with FDR < 0.05 were considered statistically significant. To identify genes associated with pseudotime progression, the associationTest function with contrastType = “end” was used to test for differential expression between trajectory endpoints. Smoothed gene-expression patterns were predicted using predictSmooth with 50 points per trajectory, and visualized as heatmaps ordered by peak expression timing. Model convergence was assessed, and only genes with successfully converged models were included in downstream analyses. Gene Ontology analysis of biological processes (GO-PB) was performed using ShinyGO (version 0.85) [25].

### 2.4. Module Score Analysis

Module score calculations were performed using the AddModuleScore function in Seurat (version 5.1) to quantify the expression of specific gene signatures across cell populations. Gene signature lists for various biological processes were obtained from curated databases. All module score calculations were performed using the RNA assay with default parameters. For each gene signature, the AddModuleScore function computed the average expression of genes in the signature subtracted by the aggregate expression of control gene sets, generating a score that reflects the relative activity of each biological process. Module scores were extracted from the Seurat object metadata for downstream statistical analysis and visualization. Normality of module scores was assessed using the Shapiro–Wilk test (α = 0.05). Comparisons between the control and ethanol groups were performed using multiple testing with false discovery rate (FDR) correction applied via the two-stage step-up method of Benjamini, Krieger, and Yekutieli. A *p*-value < 0.05 was considered statistically significant, while values between 0.05 and 0.1 were considered indicative of a modest trend.

### 2.5. Cell–Cell Communication Analysis

Cell–cell communication analysis was performed using CellChat (version 2.1.2) [26] to infer intercellular signaling networks. CellChat objects were created for each condition using the CellChatDB.human ligand-receptor interaction database. Overexpressed genes and ligand-receptor pairs were identified within each cell type using the identifyOverExpressedGenes and identifyOverExpressedInteractions functions. Communication probabilities between cell types were computed using the computeCommunProb function with the truncated mean approach (trim = 0.1), incorporating both secreted signaling (interaction range = 250 μm) and contact-dependent interactions (contact range = 100 μm). Weak or unreliable communications were filtered by requiring a minimum of 10 cells (min.cells = 10). Communication probabilities were aggregated at the pathway level using computeCommunProbPathway, and cell–cell communication networks were constructed with aggregateNet. Signaling role analysis was performed using netAnalysis_computeCentrality to identify dominant senders, receivers, mediators, and influencers within the communication network. For a comparative analysis between the control and alcohol groups, CellChat objects were merged using mergeCellChat, and a differential interaction analysis was conducted to identify group-specific communication patterns. Statistical significance of differential interactions was assessed using the built-in permutation test (do.stat = TRUE). Results were visualized using circle plots, heatmaps, and bubble plots to display interaction counts, signaling strengths, and pathway-specific communications.

## 3. Results

### 3.1. Chronic Alcohol Consumption Reprograms Cellular Metabolism During Enhanced Osteoclastogenesis

In our previous publication, we identified clusters and reported marker genes, differentially expressed genes (DEGs), and pathway enrichment analyses [5]. Briefly, we identified seven distinct cellular states among 15,445 cells. All macrophage clusters expressed *CD14* and *MAMU-DRA*. Terminal macrophages (Term-Mac) additionally expressed *CD36* and *C1QC*. Intermediate macrophages (Int-Mac) expressed interferon-related genes (*MX1*, *IFIT1*, *STAT2*). DC-like macrophages (DC-like Mac) expressed *CD52* and *ITGAX*. Proliferating macrophages (Proli-Mac) expressed high levels of cell cycle genes (*TOP2A*, *MKI67*, *CCNB1*). Differentiating macrophages (Diff-Mac) expressed *CD36*, and elevated *CDK6* and *AKT3*. Osteoclast precursors (OC-Pre) were defined by expression of *CDK6*, *AKT3*, and osteoclast genes *TNFRSF11A* and *OSCAR*. Finally, mature osteoclasts (OC) showed high expression of *TNFRSF11A*, *ATP6V0D2*, *NFATC1*, and *ACP5* [5]. While the prior studies provided initial insight into how alcohol consumption alters transcriptional programs across the osteoclast lineage, additional insights remain to be gathered.

**Figure 1:**
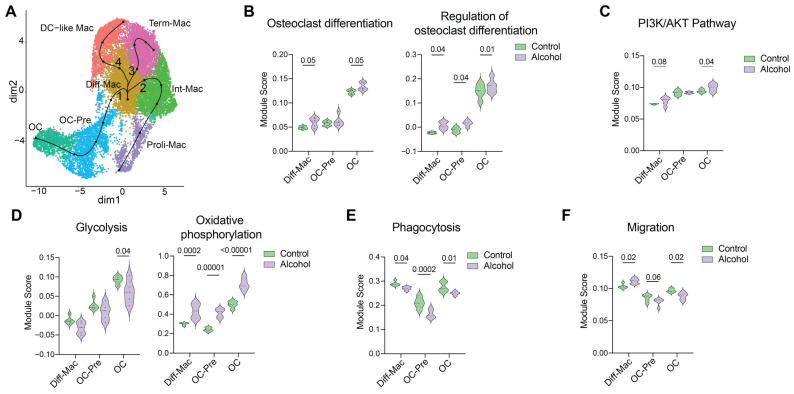
Alcohol promotes osteoclastogenesis and rewires cellular metabolism. (**A**) UMAP showing clusters and lineage trajectory curves. Module scoring performed using gene sets involved in (**B**) Osteoclast differentiation and Regulation of osteoclast differentiation, (**C**) PI3/AKT pathway, (**D**) Glycolysis and Oxidative phosphorylation, (**E**) Phagocytosis, and (**F**) Migration. Normality of module scores was assessed using the Shapiro–Wilk test (α = 0.05). For panels (**B**–**F**), multiple testing correction was performed using a FDR approach with the two-stage step-up method of Benjamini, Krieger, and Yekutieli. Adjusted *p*-values < 0.05 were considered statistically significant, while values between 0.05 and 0.1 were interpreted as indicative of a modest trend.

Here we regenerated a UMAP (Uniform Manifold Approximation and Projection) employing fitGAM/tradeSeq to model smooth, nonlinear gene-expression changes along differentiation trajectories, enabling detection of dynamic and stage-specific regulation that would be missed by the discrete comparisons previously reported [5]. Four distinct trajectory lineages were identified. Lineage 1 followed the osteoclast lineage and included Diff-Mac, OC-Pre, and mature OC. Lineage 2 comprised Diff-Mac transitioning into Int-Mac and Proli-Mac. Lineage 3 consisted of Diff-Mac progressing toward Term-Mac. Lineage 4 involved Diff-Mac differentiating into DC-like Mac (Figure 1A and Figure S1A and Table S1).

Additionally, we conducted module scoring to capture coordinated expression changes across predefined gene sets without relying on fixed differential-expression cutoffs, thereby preserving continuous variation along differentiation trajectories. Module scores of osteoclast differentiation (GO:0030316) and regulation of osteoclast differentiation (GO:0045670) were significantly increased in Diff-Mac in the alcohol group, indicating an enhanced commitment to the osteoclast lineage. Consistent with this upstream priming, module scores for these gene sets were also elevated in OC-Pre and mature OCs (Figure 1B). The PI3K/AKT pathway, which plays a critical role in osteoclastogenesis [27], was modestly enhanced in Diff-Mac but was significantly increased in mature OCs in the alcohol group compared to the controls (Figure 1C).

Given that osteoclastogenesis is an energy-demanding process [28,29,30], we next assessed module scores for glycolysis and oxidative phosphorylation. Module scores for both metabolic pathways increased along the osteoclast differentiation trajectory in both the control and alcohol groups (Figure 1D). However, oxidative phosphorylation scores were consistently higher than glycolysis across the OC lineage, irrespective of experimental condition (Figure S1B). Notably, glycolysis scores were reduced in the alcohol group compared to controls, whereas oxidative phosphorylation scores were significantly elevated following alcohol consumption (Figure 1D).

Additionally, module scores associated with phagocytic function showed a significant decrease across the differentiation trajectory in the alcohol group compared with controls (Figure 1E). Finally, the migration-related module score was highest in Diff-Mac in the alcohol group but declined progressively, reaching significantly lower levels in OC (Figure 1F). Together, module scoring indicates that alcohol consumption enhances osteoclast lineage commitment and oxidative phosphorylation, while reducing glycolytic, migratory, and phagocytic activities during in vitro osteoclast maturation.

### 3.2. Alcohol-Mediated Transcriptional Changes Lead to Early Fate Commitment Bias

**Figure 2:**
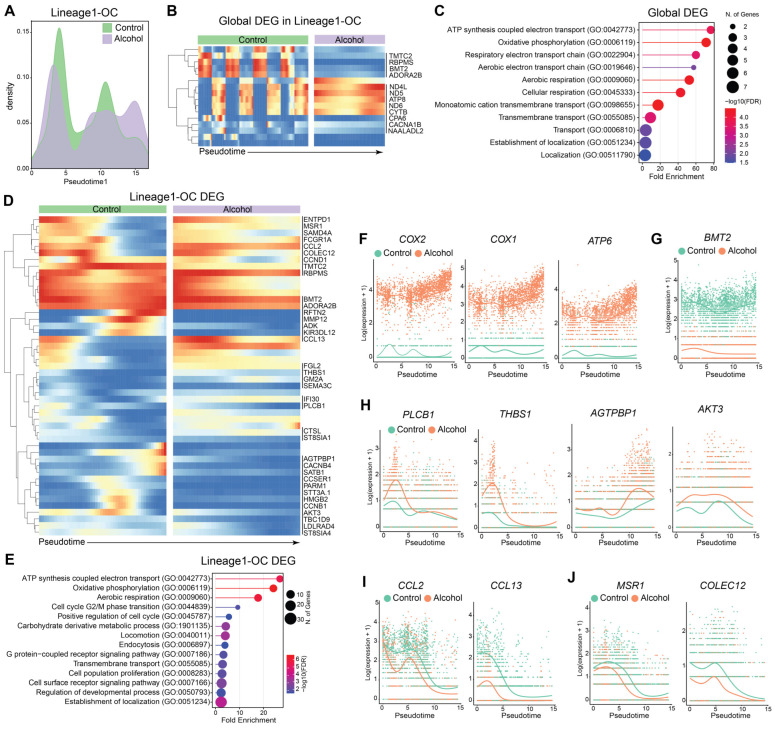
Alcohol enhances the expression of genes involved in the differentiation and metabolic adaptation of osteoclasts. (**A**) Progression plot of OC lineage. (**B**) Heatmaps depict the DEGs identified globally across OC lineage pseudotime. (**C**) Enrichment of DEGs globally dysregulated. (**D**) Heatmaps depict the DEGs identified in OC lineage across pseudotime. In (**B**,**D**), the arrow indicates the progression of differentiation. The red color shows increased expression, while the blue indicates decreased expression across the pseudotime. (**E**) Enrichment of DEGs identified along the OC lineage trajectory. Line plots showing expression of the selected DEGs along the OC lineage pseudotime, involved in (**F**) metabolism, (**G**) mTORC1 signaling, (**H**) cell cycle and proliferation, (**I**) migration, and (**J**) endocytosis.

To assess the distribution of cells along a pseudotime trajectory, we created a progression plot that reveals shifts in lineage progression and state stability. In the OC lineage, at early pseudotime, control cells showed greater accumulation, whereas alcohol cells progressed more rapidly through early states. At mid-pseudotime, alcohol consumption resulted in a broader distribution of cells along the trajectory. At late pseudotime, alcohol consumption led to increased cell accumulation or prolonged residence, suggesting stabilization of terminal states (Figure 2A). A relatively similar pattern was observed in differentiation toward Term-Mac. In contrast, the Proli-Mac lineage showed increased accumulation of cells from the alcohol group at early pseudotime, while control cells accumulated at the later pseudotime states. For the DC-like Mac lineage, early and late pseudotime distributions were comparable across groups; however, control cells showed increased density at mid-pseudotime (Figure S2A).

To further characterize transcriptional changes along pseudotime, we first identified DEGs between the alcohol and control groups regardless of differentiation trajectory (Figure 2B and Figure S2B and Table S2). The 16 DEGs identified were associated with metabolic processes, including oxidative phosphorylation and aerobic respiration (CYTB, ND5, ND4L, and ATP8), while others mapped to pathways related to transmembrane transport and localization (e.g., CACNA1B and ADORA2B), suggesting changes in cytoskeletal regulation, membrane trafficking, and cellular activation (Figure 2B,C).

We next identified lineage-specific DEGs (Figure 2D and Figure S2C and Table S2). The 105 DEGs identified in the OC lineage enriched to metabolic pathways, including ATP synthesis coupled to electron transport (COX1, CYTB, CCNB1, ND3, ND5, ND4L, ND4), oxidative phosphorylation, and aerobic respiration (e.g., CCNB1, ATP6, ATP8), indicating pronounced metabolic reprogramming along the trajectory (Figure 2D,E and Table S2). Specifically, COX1, COX2, and ATP6 were significantly overexpressed in the alcohol group (Figure 2F). Moreover, BMT2, also known as SAMTOR, a negative regulator of mTORC1 [31], showed lower expression levels across pseudotime in the alcohol group (Figure 2G). Some DEGs enriched to cell cycle-related terms, including G2/M phase transition (e.g., PLCB1) and proliferation (e.g., VCAN, NRG1, THBS1, AGTPBP1, CALCRL, AKT3, IFI30) (Figure 2D,E,H and Table S2). Finally, enrichment of pathways related to locomotion (e.g., CCL2, CCL22, PF4V1, JAML, CCL13, SEMA3C, LDLRAD4, ENPP2, CXCR4), endocytosis (e.g., SH3GL2, MSR1, COLEC12, CALCRL, CTSL), and regulation of developmental processes (e.g., TMEM176B, VCAN, MSR1, CCND1, HMGB2, LDLRAD, ENPP2, CXCR4, BCL2L11, PBX1, FGL2) highlights the extensive structural remodeling required for osteoclast maturation (Figure 2D,E and Table S2). While most of these genes were upregulated in the alcohol group, genes such as CCL2 and CCL13 were downregulated, supporting the module scores indicating diminished migratory and recruitment capacity (Figure 2I). In addition, decreased expression of MSR1 and COLEC12 further supports reduced phagocytic potential along the osteoclast lineage under alcohol exposure (Figure 2J). Together, these analyses indicate that alcohol consumption accelerates osteoclast-lineage differentiation characterized by enhanced metabolic activity, increased proliferative capacity, and coordinated structural remodeling, collectively promoting osteoclast differentiation and maturation.

### 3.3. Alcohol Amplifies Intercellular Communication During Osteoclastogenesis

**Figure 3:**
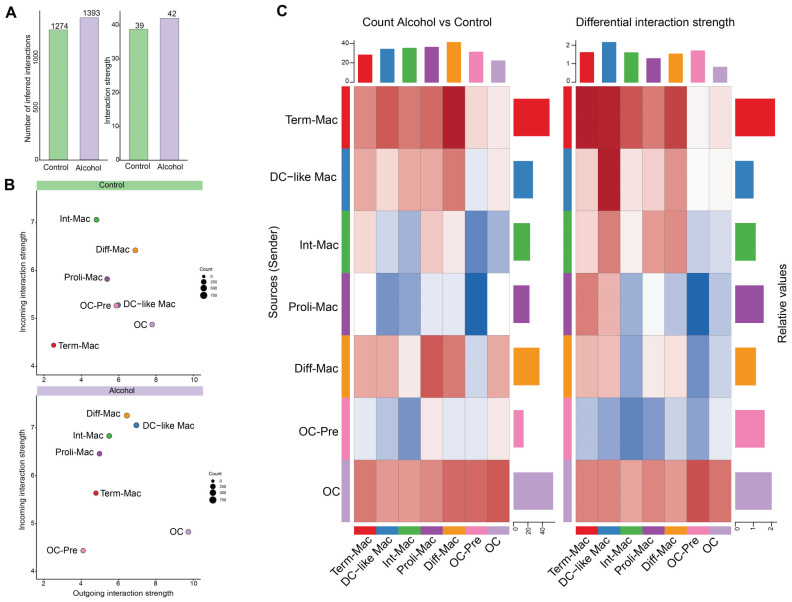
Alcohol amplifies intercellular communication, with OCs as dominant signaling hubs. (**A**) Number and strength of interactions between the control and alcohol group. (**B**) The scatter plot illustrates the incoming and outgoing interaction strengths for each cluster in the control and alcohol groups. (**C**) The heatmaps depict the differential count (**left**) and strength (**right**) of communication between cells in the sender (*y*-axis) or receiver (*x*-axis) role in each cluster. In the C heatmap, higher red intensity indicates more or stronger signaling in alcohol compared to the control, and higher blue intensity indicates fewer or weaker signaling in alcohol compared to the control.

We applied CellChat to investigate intercellular communication among cell populations, particularly signaling dynamics along the osteoclast lineage. We observed more inferred interactions across all cell populations in the ethanol group, indicating potential activation of a broader set of ligand–receptor pairs and greater network complexity. This was associated with increased interaction strength, indicating higher aggregated communication probabilities (Figure 3A).

We next compared incoming and outgoing signaling strengths within clusters to assess cell-type-specific changes. Within the OC lineage, while Diff-Mac exhibited a balanced capacity for both sending and receiving signals in the control group, an increased incoming signaling strength was observed in the ethanol group (Figure 3B). At the cluster level, Diff-Mac interacted with most populations, except OC-Pre and Proli-Mac (Figure 3C). In addition, both incoming and outgoing signaling from OC-Pre were reduced in the ethanol group (Figure 3B). Specifically, OC-Pre, as receivers, became functionally disconnected from niche-derived signals, with reduced communication from macrophage subsets and increased relative input from OCs (Figure 3C). Notably, alcohol consumption increased OCs’ outgoing signaling and decreased incoming signaling, thereby identifying OCs as a dominant regulator within the network (Figure 3B). As receivers, OCs showed strong homotypic signaling but limited incoming communication from other populations, reflecting robust self-feedback and reduced dependence on external cues (Figure 3C).

Beyond alterations within the OC lineage, ethanol consumption reshaped communication networks among non-osteoclast lineage populations. Both incoming and outgoing signaling of DC-like Macs and Term-Mac were elevated in the alcohol group (Figure 3B), particularly toward other macrophage subsets, suggesting enhanced immunomodulatory function (Figure 3C). Proli-Mac, however, just exhibited increased incoming signaling with ethanol consumption (Figure 3B,C).

Together, the increased number and strength of interactions observed in the alcohol group indicate a globally amplified and more interconnected intercellular communication network. Within this rewired network, mature OCs emerged as dominant signaling hubs, with their maintenance and functional stability primarily sustained by autocrine mechanisms.

### 3.4. Alcohol Induces Fate-Instructive, Adhesion, and Matrix-Dependent Signaling in the Osteoclast Lineage

**Figure 4:**
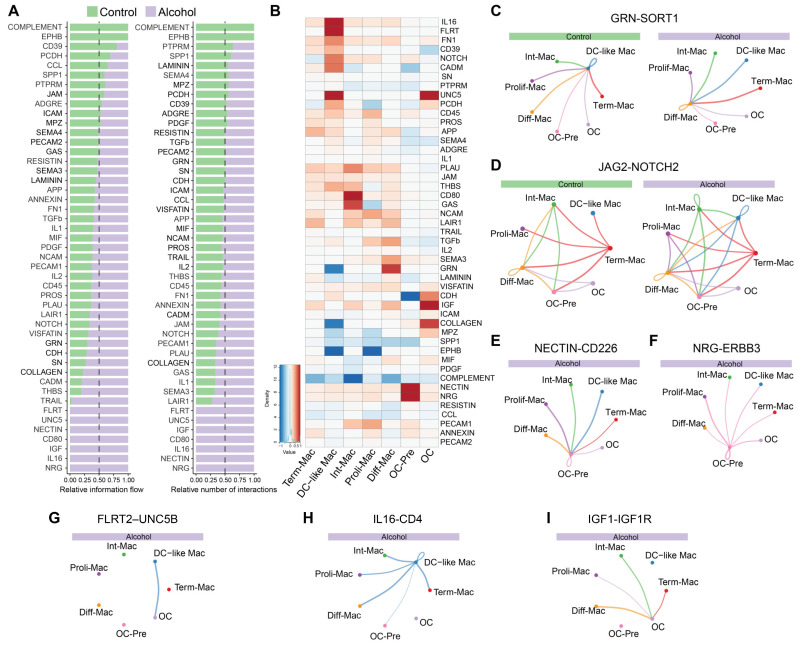
Alcohol consumption disrupts intercellular communication networks. (**A**) The heatmap of ranked pathways by relative informational flow and relative number of interactions. (**B**) The heatmap of the differential signaling strength between the groups, calculated by subtracting the strength in the alcohol group from that in the control group. Circle plots showing the network of (**C**) GRN-SORT1 (**D**) JAG2-NOTCH2, (**E**) NECTIN-CD226, (**F**) NRG-ERBB3, (**G**) FLRT2-UNC5B, (**H**) IL-16-CD4, and (**I**) IGF1-IGF1R signaling pathways. Signaling in (**E**–**I**) was detected only in the alcohol group. In (**C**–**I**), the color of the lines matches the color of the sender.

We next investigated the signaling pathways that drive intercellular communication using ranked network analysis to determine which pathways dominate the communication network. Pathways were grouped into 4 groups based on combined changes in their “Relative information flow” and “Relative number of interactions”. “Relative information flow” ranks pathways by their overall signaling strength, integrating signaling probability, gene expression, and network usage to identify those that convey the strongest communication signals. In contrast, “Relative number of interactions” ranks pathways by the frequency of ligand-receptor interactions, highlighting those used widely across the network (Figure 4A). Group 1 comprised pathways that increased in both signaling strength and interaction frequency following ethanol consumption. This group included NRG, IGF, NOTCH, UNC5, and FLRT pathways, which regulate cell fate, survival, positioning, and maturation. Immune modulatory pathways, including IL16, CD80, and CD45, were also elevated, along with adhesion and spatial organization pathways such as NECTIN, FLRT, and collagen signaling. Group 2 consisted of pathways exhibiting high information flow, but relatively modest interaction counts. These included THBS, TGFβ, Syndecan, CDH, and GRN, which mediate matrix-controlled growth factor activation, cell–cell adhesion, and intercellular remodeling. Group 3 included pathways characterized by frequent interactions but lower information flow, including SEMA3 and JAM. Finally, Group 4 encompassed pathways reduced under alcohol exposure, including EPHB, Complement, SPP1, CD39, PCDH, and PTPRM, which regulate activation, maintain immune-osteoclast balance, and stabilize cell–cell interactions (Figure 4A).

We next compared the strength of specific receptor-ligand signaling. Ethanol consumption significantly enhanced GRN-SORT1 signaling in Diff-Mac, which functioned as a signal receiver from all other populations (Figure 4B,C). Interaction of Diff-Mac with OC through TGFB1 ligand-receptor pairs was modestly increased (Figure 4B). DC-like Macs were a major source of JAG2-NOTCH2 signaling in the alcohol group (Figure 4B,D). In addition, there were signals that only emerged in the alcohol group. NECTIN-CD226 signaling, transmitted from multiple cell populations to OC-Pre, was significantly enhanced with ethanol, indicating increased adhesion (Figure 4B,E). Neuregulin (NRG)-ERBB3 signaling was also elevated with OC-Pre as the senders (Figure 4B,F). In mature OCs, alcohol slightly cadherins (CDH) signaling and preferentially enhanced FLRT2-UNC5B initiated by DC-like Mac (Figure 4B,G). IL-16-CD4 signaling was selectively initiated by DC-like Mac under ethanol consumption, providing regulatory input to both Diff-Mac and OC-Pre (Figure 4B,H). Finally, IGF1-IGF1R signaling was markedly increased in OCs, with ligand input originating from other cell populations (Figure 4B,I).

Collectively, these findings demonstrate that chronic alcohol consumption rewires intercellular communication by expanding lineage-instructive and adhesion-mediated signaling, strengthening selective fate-enforcing pathways, and maintaining spatial coordination to favor osteoclast lineage commitment and stabilization.

## 4. Discussion

Chronic alcohol consumption profoundly alters the bone marrow microenvironment through multiple mechanisms. These include disruption of growth hormone signaling, alterations in immune mediators’ levels, and epigenetic dysregulation. These changes are expected to significantly affect osteoclastogenesis [5,32,33]. Differentiation of HSPCs toward osteoclasts is initiated primarily through RANK/RANKL and M-CSF/CSF1R signaling. However, the extent of differentiation is governed by a complex network of signaling pathways [9,34,35,36,37,38,39,40,41]. We therefore sought to gain a deeper understanding of the mechanisms regulating enhanced osteoclastogenesis following chronic alcohol consumption. To that end, we assessed signaling pathways and cell–cell communication in an in vitro differentiation system. The module scoring analyses of osteoclast lineage cells revealed that chronic alcohol consumption reshapes osteoclastogenesis by reprogramming metabolic and functional states during early lineage commitment.

The increased osteoclast differentiation signatures in Diff-Mac suggest that alcohol primes progenitors. This was accompanied by enhanced PI3K/AKT module score and higher AKT3 expression in the alcohol group across pseudotime in the OC lineage. PI3K/AKT plays a critical role in osteoclastogenesis, as PI3K inhibition suppresses osteoclast formation [27]. Notably, M-CSF/CSF1R signaling promotes survival and proliferation of osteoclast progenitors through pathways including PI3K/AKT. Activation of AKT signaling promotes C/EBPα activity. This, in turn, drives transcription of essential osteoclastogenic regulators, such as *NFATc1*, *CTSK*, and *TRAP*. Together, these processes facilitate osteoclast differentiation and maturation [34,35].

During osteoclastogenesis, metabolic reprogramming provides the energy required for precursor fusion into multinucleated osteoclasts and supports bone resorption in fully differentiated cells [28,29,30,42]. Oxidative phosphorylation represents the primary bioenergetic pathway during osteoclast differentiation [43]. In contrast, glycolysis is particularly important for sustaining the high resorptive activity of mature osteoclasts [28]. Ethanol consumption induced a metabolic shift characterized by enhanced oxidative phosphorylation and reduced glycolytic activity. Studies have shown that glycolytic upregulation occurs when mature osteoclasts engage a bone or bone-like resorptive surface [30,42]. The absence of such matrix cues in our system may therefore blunt activation of osteoclast glycolytic programs. Further, assessing gene expression along the trajectories revealed that alcohol consumption, both globally and within the osteoclast lineage, dysregulated genes involved in oxidative phosphorylation and ATP production. Downregulation of BMT2 (SAMTOR) throughout the trajectory in the alcohol group further underscores this metabolic reprogramming. SAMTOR is a negative regulator of mTORC1, a master regulator of cellular metabolism [31].

M-CSF and RANKL drive macrophage/monocyte precursors commitment toward the osteoclasts rather than a phagocytic macrophage fate [44,45]; however, mature osteoclasts still perform phagocytosis to engulf bone debris, calcium–phosphate crystals, and apoptotic bone cells [46]. This phagocytic capacity is essential for maintaining bone and marrow integrity, particularly for clearing dying osteocytes and chondrocytes embedded within mineralized matrices [46]. We also observed that alcohol substantially decreased the expression of genes involved in phagocytosis. This was evidenced by a decline in the expression of scavenger receptors MSR1 and COLEC12 [47] along the OC lineage pseudotime. This pattern suggests a decreased ability to scavenge and clear particles, debris, or apoptotic material.

The migratory capacity of cells across the lineage, however, depends on cell status. Osteoclast precursors, here Diff-Mac, require higher migration capacity to reach bone surfaces, a capacity that was enhanced by alcohol. However, in committed OC-Pre and mature OCs, it was decreased. Many matrix-sensing and sealing-zone-related pathways, such as integrin αvβ3 signaling and podosome ring organization, are fully engaged only on bone or bone-like material [48,49,50]. Thus, reduced migration signatures in mature OCs may represent a more stably adherent, resorption-primed state. This phenotype would likely manifest as increased activity only in the presence of an appropriate resorptive surface. Indeed, we previously reported that osteoclasts derived from ethanol-fed animals exhibit increased resorptive activity on a calcium-coated surface [5].

Analysis of transcriptional dynamics along the pseudotime trajectory suggests that alcohol consumption reshapes osteoclast lineage progression. Alcohol reduced cell accumulation at early pseudotime and increased residence at later stages. This pattern supports a model in which alcohol accelerates initial lineage commitment and subsequently promotes persistence of mature osteoclast states. The broadened intermediate distribution may reflect extended transitional remodeling, encompassing membrane raft assembly and coordinated changes in proliferation, RNA metabolism, and metabolic adaptation that facilitate progression toward mature osteoclasts [11]. At the molecular level, alcohol consumption along the OC lineage induced expression of genes important for cell-cycle and structural remodeling (e.g., VCAN, PLCB1, and MSR1). This suggests a coupling of metabolic activation with differentiation and cytoskeletal reorganization required prior to cell fusion [51]. Additionally, COX1 and COX2 were among the genes that were highly expressed in the alcohol group along this trajectory. COX2 is a pro-osteoclastogenic factor, as RANKL-induced COX2–dependent PGE2 production in osteoclast precursors is required to support osteoclast differentiation [52]. Together, these findings indicate that chronic alcohol consumption promotes osteoclastogenesis through early lineage priming, sustained metabolic activation, and stabilization of differentiated cells.

Osteoclastogenesis is tightly regulated by transcriptional and epigenetic programs within osteoclast precursors [34,53,54] but is also shaped by signals from cells in the bone marrow niche [55]. We observed a global increase in the number and strength of interactions. This may indicate that ethanol consumption amplified signaling complexity and coordination across the network. Alcohol consumption heightened cellular crosstalk and pathway engagement through multiple mechanisms [56]. Elevated incoming signaling in Diff-Mac suggests enhanced sensitivity to instructive cues that may accelerate or bias differentiation trajectories. OC-Pre, however, becomes functionally insulated from niche-derived signals. This pattern suggests greater reliance on pre-established transcriptional and epigenetic programs inherited from upstream Diff-Mac or signals derived from mature OCs. Concurrently, mature OCs adopt a dominant regulatory phenotype characterized by strong outgoing signaling and robust homotypic feedback. This may indicate that these cells actively reshape their microenvironment. This is a hallmark of terminal differentiation, in which fate decisions are complete, and survival and activity are largely governed by intrinsic signaling loops [57]. Indeed, RANK-induced IL-8 functions as an autocrine mediator that promotes osteoclastogenesis [58]. We also previously reported that conditioned media from osteoclast cultures derived from the alcohol group, containing enhanced IL-6 compared to controls, significantly increased granulocyte–monocyte colony formation and enhanced the capacity of these progenitors to differentiate into TRAP^+^ osteoclasts [5].

There were changes in non-osteoclast-lineage populations in our in vitro culture, including the activation of Term-Mac and DC-like Mac. These findings suggest that alcohol promotes immunomodulatory engagement within the niche. This may potentially reinforce osteoclast-supportive signaling loops. Bone marrow-resident macrophages, a heterogeneous population, produce pro-osteoclastogenic cytokines (e.g., TNF-α, IL-6, IL-1β) and anti-osteoclastogenic IL-4 and IL-10, thereby influencing osteoclast differentiation [44,46]. In addition, OsteoMACs (osteal macrophages) under osteoporotic or inflammatory conditions accumulate near resorbing osteoclasts and clear resorption byproducts, thereby coordinating remodeling [46,59,60]. In addition to macrophages, other immune cells, including Th17, Th2, Tregs, B cells, and DCs, especially during immune activation, play a role in regulating osteoclastogenesis [55,61,62].

Further analysis of signaling pathways revealed that alcohol consumption does not simply increase overall signaling. Instead, it selectively reorganizes pathway hierarchies to favor osteoclast lineage commitment and stabilization. The GRN-SORT1 signaling network was completely reshaped in the alcohol group, such that all incoming signals regulate Diff-Mac. Progranulin (encoded by *GRN*) and its receptor Sortilin (*SORT1*) are linked to amplifying pro-inflammatory responses [63]. Targeting SORT1 reduces the secretion of pro-inflammatory cytokines [64]. Additionally, the TGF-β signal was slightly enhanced in the alcohol group. TGF-β priming reprograms TNF-stimulated macrophages towards osteoclasts and is required for TNF-driven inflammatory bone resorption in vivo [65].

In OC-Pre, alcohol selectively strengthened pathways associated with contact-dependent signaling and intercellular communication, including NECTIN-CD226. This signaling, however, requires further mechanistic study in osteoclasts; it initiates cell–cell junction formation and activates AKT signaling [66]. In addition, NRG1-ERBB3 signaling, which modulates proliferation and differentiation of target cells [67], was enhanced in OC-Pre.

Alcohol consumption preferentially enhanced CDH3 (P-cadherin) signaling, which is a classical calcium-dependent cell adhesion molecule in mature OCs. Survival and metabolic support pathways, including IGF, were also enhanced. IGF-I, which was sent by most cells to OCs, indicates that alcohol induces paracrine IGF signaling to support osteoclast function. It was shown that IGF-I promotes osteoclast differentiation and is required for normal osteoblast–osteoclast coupling [68]. In addition, DC-like Macs provide the ligand of the FLRT2-UNC5B signal to OCs. UNC5B is expressed in osteoclast precursors and mature osteoclasts. Netrin-1 binding to UNC5B typically inhibits osteoclast multinucleation by downregulating RANKL-induced Rac1 activation. FLRT2 competes with Netrin-1 for binding to UNC5B. When FLRT2 is absent, UNC5B becomes saturated with Netrin-1, leading to excessive inhibition of multinucleation [69]. IL-16, which was differentially overexpressed in DC-like Mac, can directly induce monocytes to differentiate into TRAP^+^ cells by activating p38 and JNK/MAPK and upregulating NFATc1 and cathepsin K [70]. Similarly, JAG2-NOTCH2 was derived by DC-like Mac. NOTCH2 is expressed on osteoclasts and promotes osteoclast differentiation [71].

While our study provides important insights into intercellular communication during osteoclast differentiation, it is not without limitations. The most notable is the use of an in vitro differentiation system on a non-resorptive surface. This in vitro system was valuable for dissecting mechanisms of cell communication. However, it cannot fully recapitulate the complexity of the in vivo bone marrow niche. For example, it does not capture biomechanical forces, vascularization, and systemic hormonal influences. Therefore, future studies will incorporate in vivo cells and bone or bone-like substrates to perform mechanistic perturbation assays to validate key signaling pathways involved in lineage progression communication networks. Additionally, the signaling pathways discussed in the manuscript represent computationally inferred interactions. These pathways are predicted to be regulatory mechanisms associated with alcohol exposure, and further experimental studies will be necessary to validate their mechanistic roles.

## 5. Conclusions

Our integrative analyses of single-cell, trajectory, and communication networks reveal that chronic alcohol consumption profoundly rewires osteoclast lineage progression by promoting early fate priming of macrophage precursors, selectively insulating lineage-committed osteoclast precursors from niche-derived signals, and stabilizing mature osteoclasts through autocrine, adhesion-mediated, and metabolically supported feedback mechanisms. These findings provide a mechanistic framework linking alcohol-induced alterations in hematopoietic differentiation, metabolic adaptation, and intercellular signaling to enhanced osteoclastogenesis and skeletal dysfunction. Importantly, this work highlights early lineage stages as critical regulatory checkpoints and identifies signaling pathways that may represent therapeutic targets to mitigate alcohol-associated bone loss.

## Data Availability

All relevant data are included in the manuscript and its Supporting Information Files. The raw data supporting the study’s findings can be obtained from the corresponding authors upon reasonable request.

## Supplementary Materials

The following supporting information can be downloaded at: https://www.mdpi.com/article/10.3390/biology15070527/s1. Figure S1: Defining trajectory lineages and impact of alcohol on osteoclast lineage; Figure S2: Alcohol impacts the differentiation of non-osteoclast lineages. Table S1: Full list of genes used for defining trajectory lineages; Table S2: Full list of global and lineage-specific DEGs.

## Abbreviations

The following abbreviations are used in this manuscript:

scRNA-seq	Single-cell RNA sequencing
HSPC	Hematopoietic stem and progenitor cell
M-CSF	Macrophage colony-stimulating factor
RANKL	Receptor activator of nuclear factor κB ligand
GAM	Generalized additive model
FDR	False discovery rate
GO-PB	Gene Ontology Biological processes
DEG	Differentially expressed gene
Diff-Mac	Differentiating macrophage
OC-Pre	Osteoclast Precursor
OC	Mature osteoclast
Int-Mac	Intermediate macrophage
Proli-Mac	Proliferating macrophage
Term-Mac	Terminally differentiated macrophage
DC-like Mac	Dendritic-like macrophage
NRG	Neuregulin
CDH	Cadherins
OsteoMAC	Osteal macrophage
