# Supplementary material for: Chronic Alcohol Consumption Reprograms Osteoclast Lineage Communications to Promote Osteoclastogenesis
Source: Biology (Basel). 2026 Mar 26;15(7):527. doi: 10.3390/biology15070527 (PMC13072430; doi:10.3390/biology15070527)
Supplement: Supplementary file 1 [file biology-15-00527-s001.zip › Figure S1.pdf]

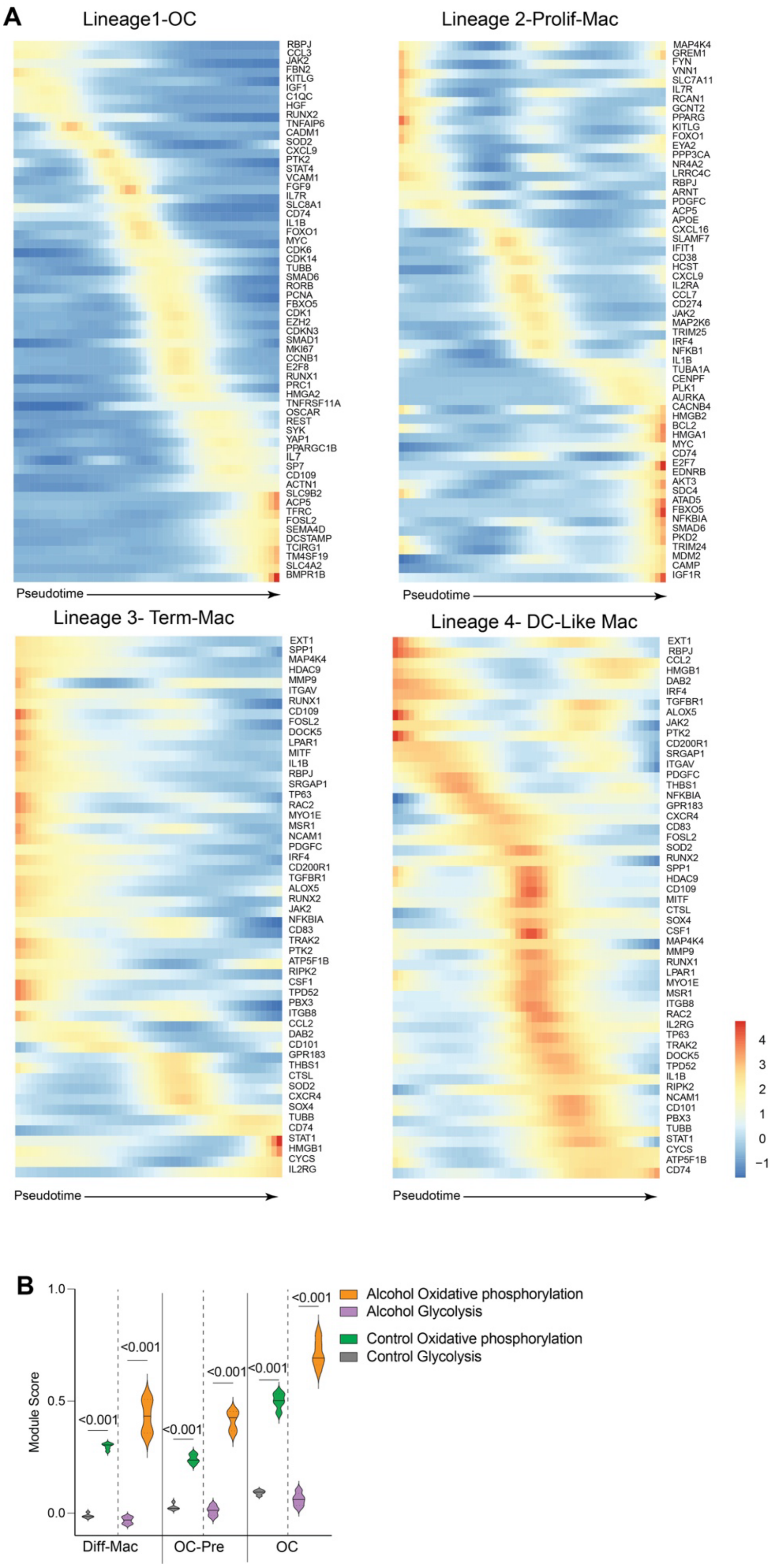

A
Lineage1-OC
RBPJ
CCL3
JAK2
FBN2
KITLG
IGF1
C1QC
HGF
RUNX2
TNFAIP6
CADM1
SOD2
CXCL9
PTK2
STAT4
VCAM1
FGF9
IL7R
SLC8A1
CD74
IL1B
FOXO1
MYC
CDK6
CDK14
TUBB
SMAD6
RORB
PCNA
FBXO5
CDK1
EZH2
CDKN3
SMAD1
MKI67
CCNB1
E2F8
RUNX1
PRC1
HMGA2
TNFRSF11A
OSCAR
REST
SYK
YAP1
PPARGC1B
IL7
SP7
CD109
ACTN1
SLC9B2
ACP5
TFRC
FOSL2
SEMA4D
DCSTAMP
TCIRG1
TM4SF19
SLC4A2
BMPR1B
Pseudotime
Lineage 2-Prolif-Mac
MAP4K4
GREM1
FYN
VNN1
SLC7A11
IL7R
RCAN1
GCNT2
PPARG
KITLG
FOXO1
EYA2
PPP3CA
NR4A2
LRRC4C
RBPJ
ARNT
PDGFC
ACP5
APOE
CXCL16
SLAMF7
IFIT1
CD38
HCST
CXCL9
IL2RA
CCL7
CD274
JAK2
MAP2K6
TRIM25
IRF4
NFKB1
IL1B
TUBA1A
CENPF
PLK1
AURKA
CACNB4
HMGB2
BCL2
HMGA1
MYC
CD74
E2F7
EDNRB
AKT3
SDC4
ATAD5
FBXO5
NFKBIA
SMAD6
PKD2
TRIM24
MDM2
CAMP
IGF1R
Pseudotime
Lineage 3- Term-Mac
EXT1
SPP1
MAP4K4
HDAC9
MMP9
ITGAV
RUNX1
CD109
FOSL2
DOCK5
LPAR1
MITF
IL1B
RBPJ
SRGAP1
TP63
RAC2
MYO1E
MSR1
NCAM1
PDGFC
IRF4
CD200R1
TGFBR1
ALOX5
RUNX2
JAK2
NFKBIA
CD83
TRAK2
PTK2
ATP5F1B
RIPK2
CSF1
TPD52
PBX3
ITGB8
CCL2
DAB2
CD101
GPR183
THBS1
CTSL
SOD2
CXCR4
SOX4
TUBB
CD74
STAT1
HMGB1
CYCS
IL2RG
Pseudotime
Lineage 4- DC-Like Mac
EXT1
RBPJ
CCL2
HMGB1
DAB2
IRF4
TGFBR1
ALOX5
JAK2
PTK2
CD200R1
SRGAP1
ITGAV
PDGFC
THBS1
NFKBIA
GPR183
CXCR4
CD83
FOSL2
SOD2
RUNX2
SPP1
HDAC9
CD109
MITF
CTSL
SOX4
CSF1
MAP4K4
MMP9
RUNX1
LPAR1
MYO1E
MSR1
ITGB8
RAC2
IL2RG
TP63
TRAK2
DOCK5
TPD52
IL1B
RIPK2
NCAM1
CD101
PBX3
TUBB
STAT1
CYCS
ATP5F1B
CD74
Pseudotime
4
3
2
1
0
−1
B
1.0
0.5
0.0
Module Score
<0.001
<0.001
<0.001
<0.001
<0.001
<0.001
Diff-Mac
OC-Pre
OC
Alcohol Oxidative phosphorylation
Alcohol Glycolysis
Control Oxidative phosphorylation
Control Glycolysis

Figure S1- Defining trajectory lineages and impact of alcohol on osteoclast lineage. **A**) Heatmap of selected genes across pseudotime utilized by Slingshot to delineate the trajectories for OC, Prolif-Mac, Term-Mac, and DC-like Mac lineages. The full list of genes is in Table S1. **B**) Re-graphing data shown in Figure 1D to compare the scores of Glycolysis and Oxidative phosphorylation modules within each cluster, across the control and alcohol groups.
