# Supplementary material for: Chronic Alcohol Consumption Reprograms Osteoclast Lineage Communications to Promote Osteoclastogenesis
Source: Biology (Basel). 2026 Mar 26;15(7):527. doi: 10.3390/biology15070527 (PMC13072430; doi:10.3390/biology15070527)
Supplement: Supplementary file 1 [file biology-15-00527-s001.zip › Figure S2.pdf]

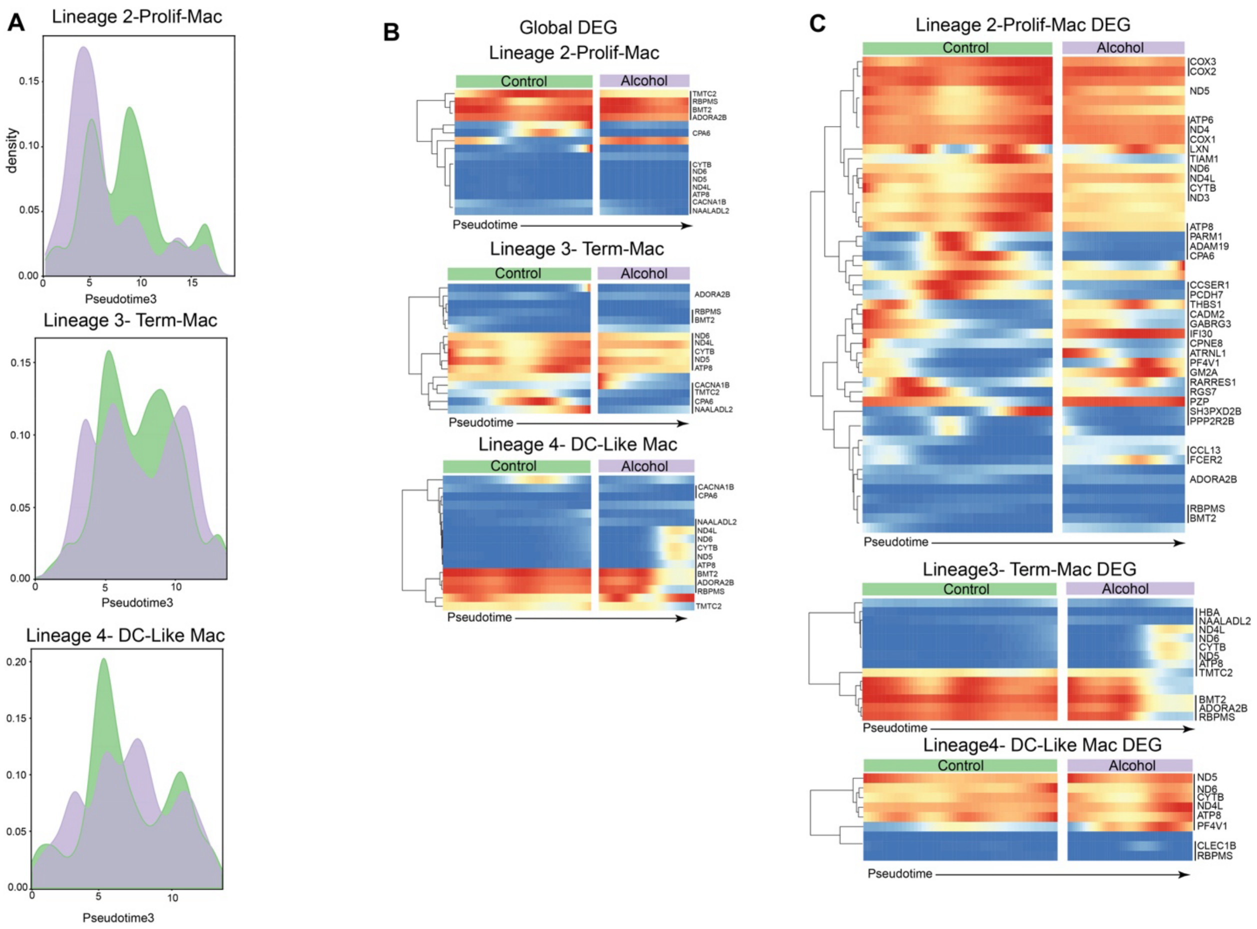

Figure S2- Alcohol impacts the differentiation of non-osteoclast lineages. A) Progression plot of Prolif-Mac, Term-Mac, and DC-like Mac lineages. **B**) Heatmaps depict the DEGs identified globally across pseudotime of Prolif-Mac, Term-Mac, and DC-like Mac lineages. **C**) Heatmaps depict the DEGs identified specifically to Prolif-Mac, Term-Mac, and DC-like Mac lineages across pseudotime. In B and C, the arrow indicates the progression of differentiation. The red color shows increased expression, while the blue indicates decreased expression across the pseudotime.
